# Development and Validation of a Radiomics Nomogram for Differentiating Pulmonary Cryptococcosis and Lung Adenocarcinoma in Solitary Pulmonary Solid Nodule

**DOI:** 10.3389/fonc.2021.759840

**Published:** 2021-11-09

**Authors:** Jiabi Zhao, Lin Sun, Ke Sun, Tingting Wang, Bin Wang, Yang Yang, Chunyan Wu, Xiwen Sun

**Affiliations:** ^1^ Department of Radiology, Shanghai Pulmonary Hospital, School of Medicine, Tongji University, Shanghai, China; ^2^ Department of Radiation Medicine, School of Basic Medical Sciences, Zhengzhou University, Zhengzhou, China; ^3^ Department of Radiology, Shanghai General Hospital, Shanghai Jiao Tong University School of Medicine, Shanghai, China; ^4^ Department of Pathology, Shanghai Pulmonary Hospital, School of Medicine, Tongji University, Shanghai, China

**Keywords:** radiomics, solitary pulmonary solid nodule, differentiate, lung adenocarcinoma, pulmonary cryptococcosis

## Abstract

**Objective:**

To establish a CT-based radiomics nomogram model for classifying pulmonary cryptococcosis (PC) and lung adenocarcinoma (LAC) in patients with a solitary pulmonary solid nodule (SPSN) and assess its differentiation ability.

**Materials and Methods:**

A total of 213 patients with PC and 213 cases of LAC (matched based on age and gender) were recruited into this retrospective research with their clinical characteristics and radiological features. High-dimensional radiomics features were acquired from each mask delineated by radiologists manually. We adopted the max-relevance and min-redundancy (mRMR) approach to filter the redundant features and retained the relevant features at first. Then, we used the least absolute shrinkage and operator (LASSO) algorithms as an analysis tool to calculate the coefficients of features and remove the low-weight features. After multivariable logistic regression analysis, a radiomics nomogram model was constructed with clinical characteristics, radiological signs, and radiomics score. We calculated the performance assessment parameters, such as sensitivity, specificity, accuracy, negative predictive value (NPV), and positive predictive value (PPV), in various models. The receiver operating characteristic (ROC) curve analysis and the decision curve analysis (DCA) were drawn to visualize the diagnostic ability and the clinical benefit.

**Results:**

We extracted 1,130 radiomics features from each CT image. The 24 most significant radiomics features in distinguishing PC and LAC were retained, and the radiomics signature was constructed through a three-step feature selection process. Three factors—maximum diameter, lobulation, and pleural retraction—were still statistically significant in multivariate analysis and incorporated into a combined model with radiomics signature to develop the predictive nomogram, which showed excellent classification ability. The area under curve (AUC) yielded 0.91 (sensitivity, 80%; specificity, 83%; accuracy, 82%; NPV, 80%; PPV, 83%) and 0.89 (sensitivity, 81%; specificity, 83%; accuracy, 82%; NPV, 81%; PPV, 82%) in training and test cohorts, respectively. The net reclassification indexes (NRIs) were greater than zero (*p* < 0.05). The Delong test showed a significant difference (*p* < 0.0001) between the AUCs from the clinical model and the nomogram.

**Conclusions:**

The radiomics technology can preoperatively differentiate PC and lung adenocarcinoma. The nomogram-integrated CT findings and radiomics feature can provide more clinical benefits in solitary pulmonary solid nodule diagnosis.

## Introduction

With the popularization of computed tomography (CT) and the application of thinner slices in chest examination, an increasing number of solitary pulmonary solid nodules (SPSNs) were screened out (1). Many pulmonary diseases may present a SPSN on chest CT in clinical practice. Lung adenocarcinoma is undoubtedly the most severe and harmful among them (2). Pulmonary cryptococcosis (PC), caused by *Cryptococcus neoformans* or *Cryptococcus gattii* infection, is challenging to differentiate from LACs when spiculated or lobulated, or other signs that signify malignant lesions (3). Therefore, pulmonary cryptococcosis presenting as a SPSN incidentally found in chest CT is frequently suspected of a malignant lesion (4–6), which causes significant distress to the patient as well as a heavy economic burden on family and society. Typically, patients in whom an uncertain SPSN was detected *via* chest CT are recommended to perform biopsy diagnosis in clinical practice (7)—a highly sensitive approach for identifying the nature of nodules (8). Nonetheless, such a method is an invasive examination and prone to false-negative results due to the limited tissue size available and the inhomogeneity and nonuniformity of tumor lesions (9). Patients, furthermore, whose biopsy is inconclusive traditionally are prescribed to fulfill regular follow-up by CT for identification, which brings unnecessary radiation exposure and may let slip optimal therapeutic window. Therefore, developing an innovative, non-invasive, and potent method is imperative to differentiate indeterminate SPSN preoperatively.

Radiomics, as an emerging technology of the medical imaging field in recent years, provides large amounts of quantitative high-throughput information of radiographic images that improve clinical decision support. In the field of chest radiology, radiomics technology was investigated broadly in predicting tumor grade, survival, and treatment response and differentiating benign from malignant lesions (10–12). Thus, it was considered as a diagnostic approach with performance approaching biopsy. However, as far as we know, there is no attempt to identify pulmonary cryptococcus and lung adenocarcinoma by quantitative imaging. Therefore, we assume that radiomics analysis based on high-dimension quantitative information can be used to distinguish between PC and LAC manifesting as a SPSN in chest CT.

Developing a CT-based diagnostic nomogram for differentiating between PCs and LACs manifesting as a solitary pulmonary solid nodule was our primary purpose of this study.

## Materials and Methods

### Patients

This retrospective study was conducted after permission from the ethics committee in Shanghai Pulmonary Hospital, and the informed consent requirement of patients was waived. The inclusion criteria were as follows: (a) pathological examination confirmed lung adenocarcinoma or pulmonary cryptococcus; (b) interval between surgery and preoperative chest CT less than 2 weeks; (c) solitary pulmonary solid nodule ≤30 mm in diameter; and (d) the thickness of the latest CT images before the surgery was not more than 1.5 mm. The cases would be excluded if they meet one of the following conditions: (a) inconclusive pathological diagnosis from an inadequate biopsy tissue specimen or bronchoscopy; (b) impure-solid nodules (pure ground-glass nodules or solid nodules with ground-glass opacity); (c) the number of pulmonary nodules was greater than one or a primary nodule with a couple of scattered lesions; (d) obvious calcifications inside nodule; and (e) patients affecting malignant tumors.

Clinical baseline characteristics were collected from the hospital information system included age, gender, smoking history, and immune status. Patients that meet one of the following criteria will be considered as immunodeficient: (a) a history of immunosuppressive therapy (treatment with immunity inhibitor), (b) diabetes mellitus, (c) AIDS, (d) recipients with a transplanted organ, (f) severe respiratory system limitation, or (g) other systemic diseases (such as lupus) and receiving steroids therapy (13–15).

### CT Image Acquisition

The chest images of the current study were acquired using one of the two CT scanners: (a) the Brilliance 40 scanner (Philips, Netherlands) with a protocol of 120 kVp tube energy and 200 mAs tube current; the parameters were 40 × 0.625 mm detector, 512 × 512 matrix, and 0.4 pitch; (b) the Somatom Definition AS scanner (Siemen, Germany) with a protocol of 120 kVp tube voltage and 130 mAs effective dose. The machine parameters were 64 × 0.625 mm detector, 1.0 pitch, and 512 × 512 matrix. The 1.0-mm thickness and 0.7-mm increment were used as reconstruction standards and applied to all CT images.

### Evaluation of Subjective Radiologic Signs

The review of the CT images of all cases was firstly completed by a junior radiologist (YY, with 6 years experience in thoracic radiology) and then inspected by a senior radiologist (XS, with more than 30 years in thoracic radiology); both were unaware of the actual pathological conclusion and clinical characteristics and analyzed the CT findings of each SPSN without discussion. All CT images were read with lung windows (level, −450HU; width, 1,500HU) and mediastinal windows (level, 40HU; width, 400HU). The following characteristics of each nodule in chest CT were assessed and recorded: (a) maximum diameter (the longest diameter of the nodule in the largest axial section); (b) size (the average of the long and short axis of the lesion on the slice, in which the nodule was the largest); (c) location (upper or middle, lower); (d) shape (round or ellipse, irregular); (e) lobulation (the contour of the nodules exhibiting concave and convex); (f) spiculation (the strands around the nodule margin and without contacting the pleural surface); (g) air bronchogram (tube-like low attenuation areas within the lesion); (h) pleural retraction (retraction of the pleura toward the nodule); and (i) cavity (lower-density shadow or air chamber in the nodule). The consensus was reached by consultation when inconsistencies between the observers exist.

### Building the Clinical Model

Combining clinical features and subjective CT findings, the clinical model was established in two steps. Firstly, the comparison in category variables (semantic characteristics, smoking history, and immune status) between two sets used the chi-squared test or Fisher’s exact test; numerical variables (maximum diameter and size) used *t*-test or Wilcoxon test to assess the differences properly. Secondly, we picked up the variables whose difference between the groups was statistically significant according to the information of Akaike and established the clinical model through the stepwise backward selection of variables by multivariate logistic regression.

### Images Segmentation and Feature Extraction

The nodules’ boundaries on each cross-section were traced based on an open software *3d-slicer* (version 4.9.0, http://www.slicer.org) on the lung window background (level, −450HU; width, 1,500HU) by a radiologist (JZ) with 1 year of experience in chest radiology and reviewed by an expert radiologist (XS) with more than 30 years of experience in thoracic imaging. All radiologists were blind to the patient information, carefully avoiding vessels, bronchus, and pulmonary parenchyma and keeping an approximate distance of 1–2 mm from the nodule margin.

Radiomic features of all nodules were automatically extracted by an in-house software based on Python (version 3.7.0, http://www.python.org) using the pyradiomics package (16). To minimize the potential impact caused by the variability of imaging parameters and scan conditions of different CT scanners and lift the convergence speed of the classification model, all CT images were preprocessed with a series of methods, including images resampled, histogram discretion, and normalization based on *Z*-score before radiomics feature extraction.

### Feature Selection and Radiomics Signature Construction

To develop a radiomics signature with robustness, high relevance, and efficient performance, a three-step feature selection process was implemented in our study: (a) assessing the reproducibility and difference of radiomics features; (b) selecting the most relevant and filtering redundant radiomics features between PC groups and LAC groups; and (c) building radiomics signature with the optimal subset of features.

Firstly, to select features insensitive to segmentation for improving the reproductivity of the model, the intra-class correlation coefficients (ICCs) were chosen as the criterion for evaluation. A radiologist (KS) aimlessly picked 30 CT images (15 PC and 15 LAC) from the training cohort and manually drew the contours of nodules independently. ICC values of more than 0.75, which means good reliability, were retained for the following analysis. Secondly, in the present study, we used the max-relevance and min-redundancy (mRMR) algorithm to rank the radiomics feature according to their correlation and redundancy between PC and LAC. Ultimately, the top-ranking features were reserved for constructing radiomics signature by calculating mutual information. Thirdly, to select the combination of the most helpful features in distinguishing between PC and LAC to optimize the predictive performance of the model, the least absolute shrink and selection operator (LASSO), an approach that calculates the regression coefficients and successively shrinks them to avoid overinflation, was implemented with fivefold cross-validation for the optimization process. Furthermore, we applied the Mann–Whitney *U* test to examine the statistical significance between the two cohorts in radiomics signature.

### Construction and Evaluation of the Classification Nomogram

To screen out the independent predictive factors for differentiating LAC from PC lesions in clinical features, radiologic signs, and radiomics signature, the multivariate logistic regression analysis was performed in the above variables. Finally, the variables with a significant statistical difference were reserved and used to construct the radiomics nomogram model.

The Hosmer–Lemeshow test was implemented to assess whether the information from variables in the nomogram was extracted and integrated by the model adequately. Furthermore, calibration curve analysis was plotted to visualize the predictive model performance. In order to evaluate the individual predictive ability of the three models, the receiver operating characteristic (ROC) curve analysis was drawn. The evaluation index of predictive performance, such as AUC, sensitivity, specificity, accuracy, NPV, and PPV, were calculated in the training and test sets, respectively. We utilized the Delong test to identify whether the difference of the AUCs from various models reaches statistical significance. Decision curve analysis (DCA) was delineated to directly present the net benefits in clinical practice with different risk thresholds. Furthermore, the net reclassification index (NRI) of the three models was calculated to compare their prediction performance.

### Statistical Analysis

ALL statistical analysis was performed using R statistical software (version 3.3.1), Python (version 3.7.0), or SPSS 20 (IBM, Armonk, NY, USA). The ROC analyses and LASSO were performed using the “sklearn” package in Python. Package “pymrmr” was used to execute the mRMR algorithm. The nomogram and calibration curve analysis was completed by “rms”, and DCA was completed by “rmda” in R programming. NRI and Delong test were calculated by the package “nricens” and “pROC”, respectively. The AUC cutoff values were established by calculating the maximal Youden index (sensitivity + specificity − 1). All statistical analyses were two-sided, and significance was set at *p* < 0.05.

## Results

### Patient Demographics

Between January 2011 and December 2020 in our hospital, we enrolled 1,482 patients (213 PC and 1,269 LAC) who fulfilled the inclusion criteria. Because the sample size of PC is much smaller than that of LAC, we adopted the 1:1 propensity score matching (PSM) approach to balance the size of the number of two cases based on age and gender. Finally, 426 patients (213 PC and 213 LAC) were matched and split into the training (151 PC and 147 LAC) and test (62 PC and 66 LAC) sets in a 7:3 ratio for subsequent research. The clinicopathological factors and the recorded CT features are displayed in **Table 1**.

**Table 1:** Clinical characteristics and radiologic signs from SPSNs in LAC and PC groups.

Variates	Train cohort (*n* = 298)		*p*-value	Test cohort (*n* = 128)		*p*-value
	PC (*n* = 151)	LAC (*n* = 147)		PC (*n* = 62)	LAC (*n* = 66)	
Gender			.308			.089
Female	60 (39.7%)	67 (45.6%)		20 (32.26%)	31 (46.97%)	
Male	91 (60.3%)	80 (54.4%)		42 (67.74%)	35 (53.03%)	
Smoking status			.671			.692
Never smoked	130 (86.09%)	129 (87.76%)		51 (82.26%)	56 (84.85%)	
Former or current smoker	21 (13.91%)	18 (12.24%)		11 (17.74%)	10 (15.15%)	
Immune status			.070			.894
Immunocompetent	134 (88.74%)	139 (94.56%)		54 (87.10%)	58 (87.88%)	
Immunocompromised	17 (11.26%)	8 (5.44%)		8 (12.90%)	8 (12.12%)	
Maximum diameter	15.54 ± 4.39	17.97 ± 6.14	.001*	15.28 ± 4.46	18.08 ± 5.86	.003*
Age	56.91 ± 10.13	56.76 ± 10.71	.749	55.32 ± 8.20	56.71 ± 11.27	.448
Size	14.10 ± 5.23	15.90 ± 5.13	.004*	13.82 ± 4.19	16.07 ± 5.73	.016*
Location			.202			.082
Upper or middle	70 (46.36%)	79 (53.74%)		30 (48.39%)	42 (63.64%)	
Lower	81 (53.64%)	68 (46.26%)		32 (51.61%)	24 (36.36%)	
Shape			.387			.592
Round or ellipse	85 (56.29%)	90 (61.22%)		33 (53.23%)	32 (48.48%)	
Irregular	66 (43.71%)	57 (38.78%)		29 (46.77%)	34 (51.52%)	
Lobulation			<.001*			.001*
Absent	51 (33.77%)	12 (8.16%)		18 (29.03%)	4 (6.06%)	
Present	100 (66.23%)	135 (91.84%)		44 (71.97%)	62 (93.94%)	
Spiculation			.113			.416
Absent	67 (44.37%)	52 (35.37%)		23 (37.10%)	20 (30.30%)	
Present	84 (55.63%)	95 (64.63%)		39 (62.90%)	46 (69.70%)	
Air bronchogram			.353			.003*
Absent	115 (76.16%)	105 (71.43%)		52 (83.87%)	40 (60.61%)	
Present	36 (23.84%)	42 (28.57%)		10 (16.13%)	26 (39.39%)	
Pleural retraction			<.001*			.001*
Absent	131 (86.75%)	78 (53.06%)		47 (75.81%)	31 (46.97%)	
Present	20 (13.25%)	69 (46.94%)		15 (24.19%)	35 (53.03%)	
Cavity			.705			.956
Absent	147 (97.35%)	145 (98.64%)		59 (96.77%)	65 (98.48%)	
Present	4 (2.65%)	2 (1.36%)		2 (3.23%)	1 (1.52%)	

Differences were assessed by the Wilcoxon Rank Sum test or Pearson chi-square test. SD, standard deviation. *p < 0.05.

After matching, a total of 426 patients were enrolled in the present study. The training cohort contained 298 patients—171 males (age range, 24–81 years; mean age, 56.46 ± 10.93 years) and 127 females (age range, 28–82 years; mean age, 57.11 ± 10.02 years). The test cohort had 128 patients—77 males (age range, 32–78 years; mean age, 57.98 ± 11.19 years) and 51 females (age range, 38–77 years; mean age, 53.97 ± 8.77 years). The maximum diameter, size, lobulation, and pleural retraction had significant statistical differences between the PC and LAC in the training cohort (*p* < 0.05, **Table 1**).

Multivariable analysis is presented in **Table 2** and showed that the maximum diameter (odds ratio [OR], 1.075; 95% confidence interval [CI], 1.032–1.120; <0.001), size (OR, 1.069; 95% CI, 1.022–1.118; =0.003), lobulation (OR, 0.174; 95% CI, 0.088–0.344; <0.001), and pleural retraction (OR, 0.173; 95% CI, 0.097–0.306; <0.001) were independent predictive factors for the clinical model.

**Table 2:** Results of logistic regression analysis for clinical features.

Variates	Univariate logistic regression		*p*-value	Multivariate logistic regression		*p*-value
	OR	95% CI		OR	95% CI	
Maximum diameter	1.075	1.032–1.120	<.001*	1.059	1.011–1.108	.016*
Size	1.069	1.022–1.118	.003*	—	—	—
Lobulation	0.174	0.088–0.344	<.001*	0.205	0.099–0.424	<.001*
Pleural retraction	0.173	0.097–0.306	<.001*	0.199	0.109–0.361	<.001*

*p < 0.05.

### Radiomics Model Construction

**Figure 1:**
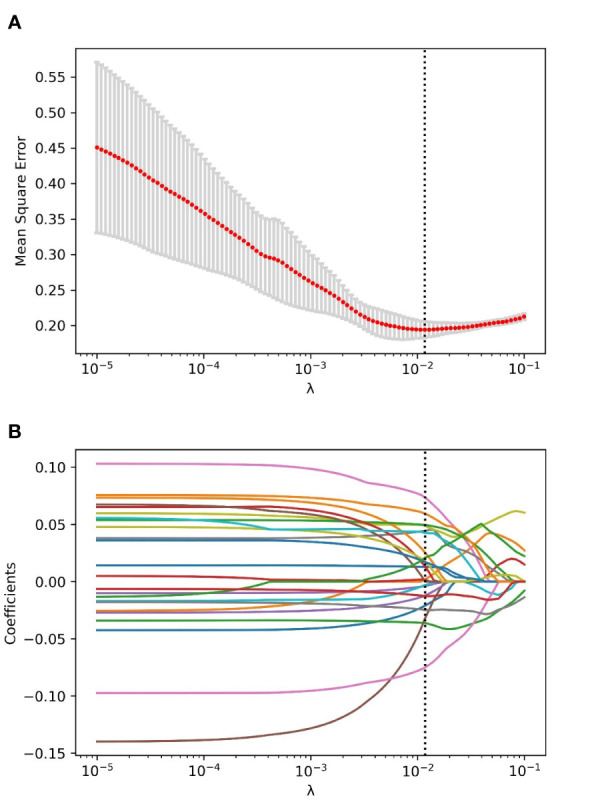
The process of radiomics features selection using the LASSO algorithm. **(A)** The relationship between mean square error (MSE) and parameter (*λ*) was visualized. As the parameter (*λ*) increased, the MSE decreased gradually. When the value of *λ* was 0.0118, which was supposed to be the optimal parameter based on fivefold cross-validation, the MSE reached the lowest point, and the dotted vertical line was plotted. **(B)** The coefficient profile of the radiomics features in LASSO analysis. As the parameter swelled, more and more coefficients of features were compressed to zero. When the value of *λ* was 0.0118, and log(*λ*) = −1.9293, an optimal subset with 24 non-zero features was yielded.

**Figure 2:**
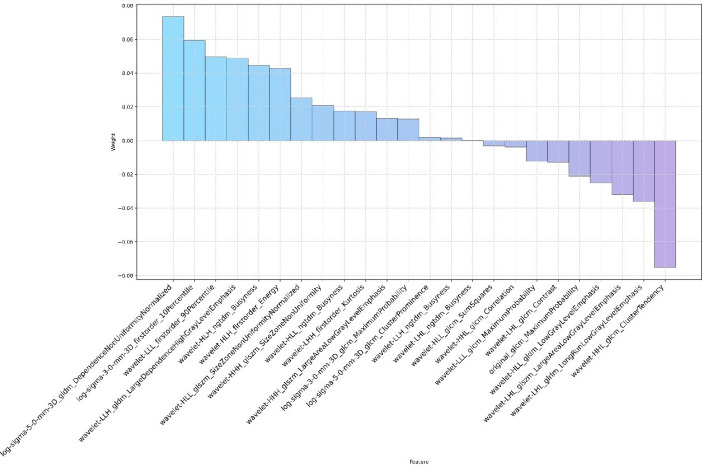
The name and coefficient of the remaining 24 radiomics features after feature selection by the LASSO method.

Through the inter- and intra-observer agreement test, 631 radiomics features had ICC values greater than 0.75 totally, which means good reproductivity. Then, the mRMR analysis was performed with 631 radiomics features (ICC > 0.75), and the top 100 highly relevant and nonredundant features were picked up for radiomics signature construction. The most optimal feature combination for distinguishing PC from LAC was calculated by the LASSO algorithm. When lambda (*λ*) was 0.0118 and log(*λ*) was −1.9293, a subset consisted of 24 radiomics features was screened out to develop a radiomics signature (**Figures 1A, B**). **Figure 2** shows the coefficients of the 24 potential predictors in order of importance.

**Figure 3:**
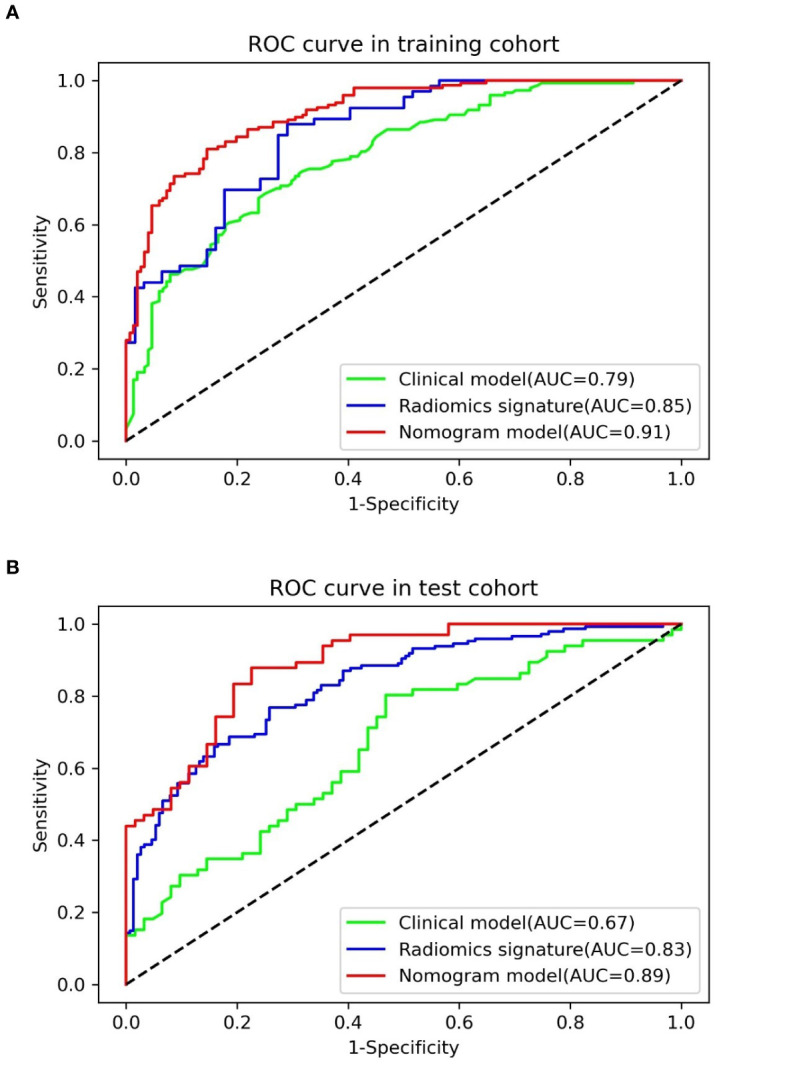
ROC curves and AUCs of three diagnostic models in the **(A)** training and **(B)** test cohorts.

The result of the Wilcoxon Rank Sum test suggested that the radiomics signature had a significant statistical difference between pulmonary cryptococcosis and lung adenocarcinoma in training and test sets (all *p* < 0.0001), which indicated that the radiomics signature had a good identification efficiency in the training (AUC = 0.8519; 95% CI, 0.7881–0.9157) and test (AUC = 0.8306; 95% CI, 0.7853–0.8759) cohorts, respectively (**Figure 3**).

### Diagnostic Nomogram Development and Assessment

**Figure 4:**
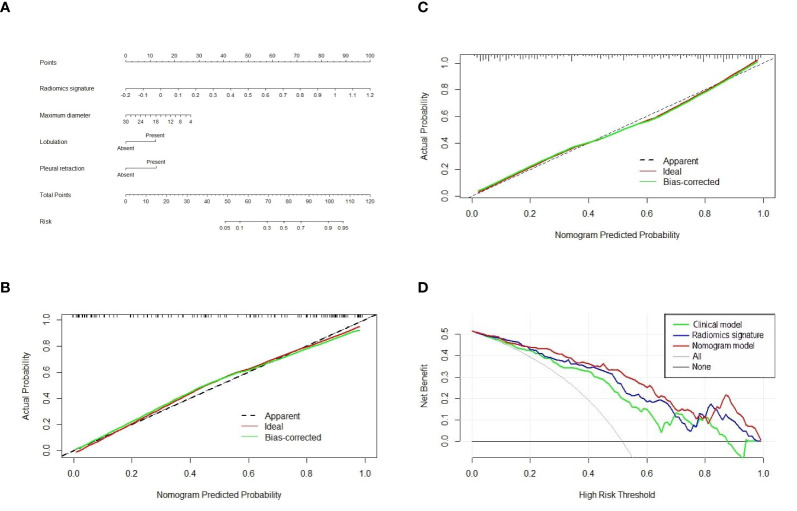
The nomogram, calibration curve, and decision curve analysis. **(A)** A nomogram incorporating radiomics signature and radiologic signs was constructed on the basis of the training set. The calibration curve of the nomogram model in the training cohort **(B)** and test cohort **(C)**. The abscissa axis represented the predictive probability by nomogram, and the vertical axis meant the actual lung adenocarcinoma probability. The ideal and bias-corrected probabilities were presented with the solid red and green lines, respectively. **(D)** Decision curve analysis of three predictive models.

In accordance with the multivariable logistic regression analysis (shown in **Table 3**), the radiomics signature (OR, 1,365.794; 95% CI, 174.8112013–10,670.895; *p* < 0.001), maximum diameter (OR, 0.904; 95% CI, 0.842–0.970; *p* = 0.005), lobulation (OR, 0.292; 95% CI, 0.128–0.670; *p* = 0.004), and pleural retraction (OR, 0.279; 95% CI, 0.142–0.549; *p* < 0.001) were statistically significantly different and independent differentiators. On the basis of these four independent factors, we developed a combined radiomics nomogram incorporating radiomics signature and clinical characteristics (**Figure 4A**).

**Table 3:** Multivariate logistic regression analysis of nomogram model.

Variates	*β*	S.E.	Wals	*p*-value	OR	95% CI
Maximum diameter	−0.101	0.36	7.912	.005*	0.904	0.842, 0.970
Size	—	—	—	—	—	—
Lobulation	−1.229	0.423	8.436	.004*	0.292	0.142, 0.549
Pleural retraction	−1.277	0.345	13.678	<.001*	0.279	0.142, 0.549
Radiomics signature	7.219	1.049	47.376	<.001*	1365.794	174.811, 10,670.895
Constant	−0.839	0.566	2.199	.138	0.432	—

CI, confidence interval; OR, odds ratio; S.E., standard error. *p < 0.05.

The calibration curve analysis was plotted and revealed an apparent connection between the actual and predicted labels (**Figures 4B, C**). The Hosmer–Lemeshow test showed that the result was statistically nonsignificant, which means fine goodness of fit.

**Table 4** presents the various indexes of diagnostic efficiency of three models in training and test cohorts, respectively. To intuitively demonstrate and compare the identification performance of these models, the ROC curves are designed in **Figure 3**. When the model was generated with clinical characteristics alone, the AUC = 0.7901 (95% CI, 0.7398–0.8403) in the training set, which was inferior to another single model including the radiomics features alone in which the AUC = 0.8519 (95% CI, 0.7881–0.9157). However, when clinical characteristics and radiomics signature were integrated into a radiomics nomogram, the combined model yields the best discrimination effectiveness in the training cohort and the AUC was increased to 0.9101 (95% CI, 0.8787–0.9416).

**Table 4:** Predictive performances of three models in the training and test cohort, respectively.

Index	Training cohort (*n* = 298)			Test cohort (*n* = 128)		
	Clinical model	Radiomics signature	Radiomics nomogram	Clinical model	Radiomics signature	Radiomics nomogram
Cutoff value	0.5279	−0.2206	0.5840	0.4473	−0.1027	0.4555
AUC (95% CI)	0.7901 (0.7398–0.8403)	0.8519 (0.7881–0.9157)	0.9101 (0.8787–0.9416)	0.6654 (0.5718–0.7591)	0.8306 (0.7852–0.8759)	0.8881 (0.8336–0.9425)
Accuracy	0.7114 (212/298)	0.7382 (220/298)	0.8154 (243/298)	0.5859 (75/128)	0.7500 (96/128)	0.8203 (105/128)
Sensitivity	0.7152 (108/151)	0.7417 (112/151)	0.8013 (121/151)	0.6290 (39/62)	0.7258 (45/62)	0.8065 (50/62)
Specificity	0.7075 (104/147)	0.7347 (108/147)	0.8299 (122/147)	0.5455 (36/66)	0.7727 (51/66)	0.8333 (55/60)
PPV	0.7152 (108/151)	0.7417 (112/151)	0.8288 (121/146)	0.5652 (39/69)	0.7500 (45/60)	0.8197 (50/61)
NPV	0.7152 (108/151)	0.7417 (112/151)	0.8013 (121/151)	0.6290 (39/62)	0.7258 (45/62)	0.8065 (50/62)

CI, confidence interval; PPV and NPV, positive and negative predictive values. Numbers in parentheses were used to calculate percentages.

In the test cohort, the AUC of nomogram fell slightly compared to the training cohort, but still kept a decent classification ability (AUC = 0.8881; 95% CI, 0.8336–0.9425; sensitivity = 0.8013; specificity = 0.8333; accuracy = 0.8203) (**Table 4**). Significant differences (nomogram model *vs*. clinical model) concerning AUCs were confirmed by the Delong test in the training set (*p* < 0.0001) and test set (*p* < 0.0001), respectively. Likewise, the improvement of differentiation capacity of radiomics nomogram in comparison to the subjective CT model had been demonstrated by NRI (training cohort: NRI = 0.2672, *p* = 0.0068 and test cohort: NRI = 0.2815, *p* = 0.0462). Finally, the DCA indicated that the nomogram model brought more clinical net benefit to patients with a SPSN compared to the single model containing clinical features alone within most of the range of the threshold probability (**Figure 4D**).

## Discussion

This study proposed a diagnostic radiomics nomogram integrating clinical characteristics and radiomics features for discriminating pulmonary cryptococcosis and lung adenocarcinoma and validated its differentiation efficiency using ROC curves, AUC, Delong test, NRI, and DCA. All assessment indicators revealed that the combined nomogram model was superior to the single model in distinguishing the PC from LAC.

Formerly, pulmonary cryptococcosis was deemed to occur in the immunodeficient population represented by AIDS, severe diabetes, and organ transplanters. However, an increasing number of studies suggested that *Cryptococcus* infection in the immunocompetent patients is not rare (13, 17). In the present research, immunocompetent patients accounted for 88.26% of the patients in the series, which was significantly higher than other studies. We speculated that this is related to the fact that we only included the patients manifested as a solitary pulmonary solid nodule, whereas immunocompromised patients presented with multiple nodules or a huge mass (14, 18–21). In addition, 62.44% (133/213) of patients in the current study were male, which matched with the previous studies (13, 17).

Distinguishing pulmonary cryptococcosis from lung adenocarcinoma is an intractable conundrum, especially when a solitary solid nodule is encountered at chest CT. In clinical practice, it may be inappropriate to wait for a couple of months (22) to substantiate whether a solitary pulmonary solid nodule is malignant or not because of the risk of metastasis of cancer cells or cryptococcal meningitis (23). Numerous studies have endeavored to explore clinical factors or subjective CT characteristics that improve the dilemma of pulmonary cryptococcosis diagnosis. Deng et al. (17) included clinical information and CT signs of 68 patients affecting cryptococcus and found no specific diagnostic indicators of pulmonary cryptococcosis. In clinical practice, serum cryptococcal antigen (sCRAG) is the common biomarker for diagnosis and disease monitoring (24). Aberg et al., however, found that the sensitivity of sCRAG in immunosuppressed populations was only 39% (25). A retrospective study (21) of 18F-FDG PET/CT findings in 42 patients affected by cryptococcosis found that pulmonary nodules in 88% of subjects manifest high FDG uptake, a sign of malignancy. The above studies were based on routine laboratory diagnosis or imaging technology in clinical practice but have not yielded encouraging conclusions.

Radiomics converts traditional medical images, applying advanced computational methodologies, into exploitable data information that cannot be captured by the naked eye and carries out high-throughput quantitative analysis on them (26–31). To our knowledge, this is the first study to classify PC and LAC manifesting a solitary pulmonary solid nodule based on advanced radiomics technology.

Univariable analysis in the current research showed that the difference of four morphologic features (maximal diameter, size, lobulation, and pleural retraction) between PC and LAC had statistical significance in the training set and were incorporated into the training set multivariable logistic regression model for further investigation. Ultimately, a clinical model consisting of maximal diameter, lobulation, and pleural retraction was constructed. Due to the spread of tumor cells in the lung interstitium, the edge of the malignant lesion was usually rough (such as lobulated or spiculated). Previous studies have shown that lesion shape helped determine benignity on CT images. Nonetheless, the identification of the semantic or morphology characteristics of the lesion on CT images by radiologists was individualized. Meanwhile, the variability among observers was considerable and nonnegligible. A previous study indicated that 20% of lung cancer nodules had smooth, rounded edges, and the nodules with bumpy contours, likewise, might be discovered in inflammatory or infectious lung disease (32). Therefore, the lesion margin (smooth, lobulated, or spiculated) was only a weak predictor of the likelihood of malignancy (33). This may account for the week or moderate diagnostic ability of the clinical model in the two datasets (AUC = 0.79, 0.67, respectively).

A three-step feature selection process screened out 24 of 1,130 radiomics features, which suggested that these 24 features were highly correlated with the distinction between PC and LAC. Nevertheless, these features were not confined to a single category but comprised one Gray Level Co-occurrence Matrix (GLCM) feature, four Laplacian of Gaussian (LoG) features, and 19 wavelet features. This finding underscores the significance of comprehensive features of the nodular region and references to the microscopic and macroscopic features of the lesion. In addition, the critical point to note the bulk of these features cannot be recognized and quantified by the naked eye, which accentuates the advantages of applying automated methodology and extraction of high-order radiomics features to aid radiologists and clinicians in lesion assessment and clinical decision-making. However, how to explain the implication of radiomics features and the biological behavior behind them remains a challenge. These features reflected the microstructure and local microenvironment of the lesion to some extent.

Wavelet features, as the majority of the radiomics signature (19/24), quantified the heterogeneity of different levels of lesions that cannot be identified by human eyes, which was supposed to have excellent performance in predicting and to play an essential role in developing radiomics signature (34). The nomogram AUC constructed from subjective radiological features was the highest (0.89), as shown in **Table 4**, which shows that the human recognition symbols and the knowledge derived from machine learning were complementary.

Finally, we admit that there were still several imperfections in this study. Firstly, as a result of the property of retrospective studies, the selection bias was inevitable in patient incorporation. Secondly, since the source of the sample in the present research was from a single hospital in China, a multi-center study with a larger dataset is needed in the future to evaluate the generalization ability and general applicability of the nomogram. Additionally, the segmentation of ROI in our study was only confined to the nodule itself, which only provided a limited intra-lesion information for differentiation. However, there were previous studies that indicated that the extra-nodule region contained massive biological information that was instrumental to predict the treatment response and overall survival (35, 36). The information may also assist to determine the nature of the nodule, which needs to extract external radiomics features of the lesion in further research to confirm.

## Conclusion

The present study formulated and verified the hypothesis that a CT-based radiomics nomogram could distinguish pulmonary cryptococcosis and lung adenocarcinoma from the population with a solitary pulmonary solid lesion. Moreover, the favorable performance of the differentiation model herein was validated, which indicated that the nomogram, as a non-invasive and quantitative analysis tool, can assist clinicians in escaping from misdiagnosis or inappropriate therapeutic options.

## Data Availability Statement

The raw data supporting the conclusions of this article will be made available by the authors, without undue reservation.

## Ethics Statement

The studies involving human participants were reviewed and approved by Shanghai Pulmonary Hospital, Tongji University School of Medicine. Written informed consent for participation was not required for this study in accordance with the national legislation and the institutional requirements. Written informed consent was not obtained from the individual(s) for the publication of any potentially identifiable images or data included in this article.

## Author Contributions

XS and CW designed the study. JZ collected and classified the data and established the models. LS did the statistical analysis. KS, TW, BW, and YY made substantial revisions to the manuscript. All authors contributed to the article and approved the submitted version.

## Funding

This work was supported by the Natural Science Foundation of Shanghai [Grant Number 19ZR1443100], clinical research project of Shanghai pulmonary hospital [Grant Number fk18007] and Science and Technology Innovation Plan of Shanghai Science and Technology Commission [Grant Number 21Y11910400].

## Conflict of Interest

The authors declare that the research was conducted in the absence of any commercial or financial relationships that could be construed as a potential conflict of interest.

## Publisher’s Note

All claims expressed in this article are solely those of the authors and do not necessarily represent those of their affiliated organizations, or those of the publisher, the editors and the reviewers. Any product that may be evaluated in this article, or claim that may be made by its manufacturer, is not guaranteed or endorsed by the publisher.
